# Sustainable Thermal Post-Processing of PLA 3D Prints: Increased Dimensional Precision and Autoclave Compatibility

**DOI:** 10.3390/jfb16090334

**Published:** 2025-09-08

**Authors:** Florina Chiscop, Carmen-Cristiana Cazacu, Dragos-Alexandru Cazacu, Costel Emil Cotet

**Affiliations:** 1 Robots and Production Systems Department, Faculty of Industrial Engineering and Robotics, National University of Science and Technology POLITEHNICA Bucharest, Splaiul Independenței 313, 060041 Bucharest, Romania; florina.chiscop@upb.ro (F.C.); costel.cotet@upb.ro (C.E.C.); 2 Education Team, PTC Eastern Europe SRL, Splaiul Independenței 319, 060044 Bucharest, Romania; acazacu@ptc.com

**Keywords:** 3D printing, thermal post-processing, solid media annealing, dimensional accuracy, autoclave compatibility, sustainable manufacturing, biodegradable polymers

## Abstract

This study investigates the thermal properties and sterilization efficacy of polylactic acid (PLA) components fabricated via fused deposition modeling (FDM), focusing on PLA’s compatibility with autoclave sterilization protocols. While PLA is extensively recognized for its biobased and biodegradable characteristics, its limited thermal stability has traditionally restricted its application in high-temperature sterilization settings, such as in medical contexts. In our research, we examined three distinct specimen geometries—cylindrical, rectangular, and curved—subjecting them to thermal post-processing through constrained annealing, employing salt or silicone as the embedding medium. Following this process, we exposed the specimens to elevated temperatures, simulating typical sterilization conditions. The outcomes indicated that the annealed PLA specimens exhibited dimensional stability at temperatures exceeding 170 °C, thereby demonstrating their viability for steam sterilization procedures. To translate these findings into practical applications, we selected a small, complex geometrically relevant component, the Easy Bone Collector (EBC) shell, for autoclave testing at 134 °C. Post-sterilization, the part successfully retained its shape and functionality, indicating that, with appropriate thermal conditioning, PLA can be effectively utilized to manufacture cost-efficient, autoclavable components suitable for medical use. These results reveal a promising and sustainable approach to producing reusable, sterilization-compatible PLA devices, particularly in low-volume or single-use applications where biodegradability is advantageous.

## 1. Introduction and Background

Polylactic acid (PLA) is a bio-based thermoplastic widely used in fused deposition modeling (FDM), owing to its low processing temperature and biocompatibility [1]. However, FDM-printed PLA parts frequently suffer from dimensional inaccuracy and orthotropic/anisotropic behavior due to layer-wise deposition, thermal shrinkage/warpage, and limited through-thickness bonding [2,3]. While practical design rules and slicer-level compensations (e.g., axis shrinkage factors) can mitigate errors, they do not fully address shape stability at elevated temperatures or under sterilization [3,4].

Thermal post-processing (annealing) is a solvent-free route to enhance PLA performance by relieving internal stresses, improving interlayer fusion, and increasing ordering/crystallinity, thereby raising thermal stability and strength [1,5,6,7,8,9]. However, if heat is not delivered uniformly, annealing may introduce unwanted distortion (shrinkage/warping), so constrained setups and fixtures are explored to preserve geometry [1,6,7,10,11,12]. Recent studies show significant gains after annealing and highlight the need to balance conditioning against dimensional accuracy [5,6,7,13,14].

Early investigations documented direction-dependent behavior in PLA prints, with build orientation strongly influencing stiffness and strength. For medical-use geometries, Grădinaru et al. quantified how orientation and thermal conditioning affect compressive properties [15]. More recently, Li et al. reported orthotropic elastic/plastic responses of FDM-PLA, reinforcing the need to control both the geometry and the process when dimensional and mechanical fidelity are required after post-processing [16]. These findings motivate our focus on preserving dimensional accuracy while minimizing property drift during heat exposure.

Complementary to thermal treatment, surface and composition changes can tailor functional and thermal behavior. Alexandrescu et al. showed that adding silver particles to PLA alters surface morphology and chemistry (SEM/FTIR and wettability), enabling functional surfaces without fundamentally changing printing workflows [17]. Sustainable composite pathways—Hannachi et al. (olive-kernel/alfa fibers) and Blanzeanu et al. (chitosan–PLA from seafood waste)—demonstrate that bio-fillers can improve properties while aligning with circular economy goals [18,19]. A recent review by Tahir and Seyam synthesizes plant-fiber-reinforced PLA strategies for greening FDM, highlighting gains in stiffness/heat resistance alongside processing trade-offs (dispersion, interfacial bonding) that impact dimensional control [20]. Our study remains material-agnostic (neat PLA) and targets a practical thermal pathway that can coexist with such material strategies.

Beyond laboratory coupons, PLA is increasingly assessed in harsher or application-like media. Csótár et al. evaluated the mechanical performance and chemical stability of PLA-based reinforcements in concrete environments, underscoring that environmental exposure drives property changes and that processing/conditioning history is pivotal for durability [21]. Although distinct from steam sterilization, this evidence supports the general premise that controlled thermal history (including annealing) is key for dimensional and mechanical stability of PLA in real-world use.

Application-level constrained annealing. Focusing on functional devices, Wijnbergen et al. investigated tough-PLA prosthetic sockets and showed that annealing reduced deformation and increased mechanical strength in application-relevant geometries [22]. This application-level evidence aligns with our aim to quantify dimensional change (Δ, %Δ) on both generic features and clinically relevant components after constrained annealing and steam exposure.

A key application driver is steam sterilization (121–134 °C). Standard PLA typically softens above its glass transition, and moisture/heat can degrade properties, making autoclave cycles challenging for as-printed parts [5,23,24,25]. Nevertheless, multiple reports indicate that appropriately conditioned PLA—via material choice, geometric design, and pre-annealing—can better withstand autoclaving with reduced geometric change and acceptable strength retention [5,10,23,24,25,26,27]. Recent work even demonstrates fully autoclavable PLA devices for bioprocessing applications when geometry and processing are carefully engineered [26].

### 1.1. Gap and Objective

Although annealing of PLA is widely studied, there is still limited bench-practical, head-to-head evidence on constrained-annealing media that a user can implement to preserve dimensions while enabling a 134 °C steam cycle. In particular, a systematic comparison between salt embedding and room-temperature-vulcanizing (RTV) silicone embedding for FDM-PLA—coupled to quantitative dimensional metrics on generic features and on a clinically relevant component—remains under-documented [5,10,12,23,24,25,27].

Specific gap and novelty: prior constrained approaches chiefly use rigid molds or granular media; a conformal, non-adherent resin medium has not been systematically evaluated under an identical printing baseline and explicitly tied to a 134 °C steam cycle. Our novelty lies precisely in this “annealing-in-resin” route (RTV encapsulation) and its head-to-head comparison with salt, reported with dimensional metrics (Δ, %Δ) [5,10,12,23,24,25,27].

Applied motivation: in practice, for FDM-PLA devices that must be autoclaved at 134 °C, choosing a constrained-annealing medium (salt vs. resin/RTV) is an immediate, shop-floor decision. In the absence of comparative data on the same printing baseline, users cannot anticipate dimensional drift across the print → anneal → steam sequence, even though the literature indicates general benefits of annealing [5,10,12,23,24,25,27].

### 1.2. Hypothesis and Scope

We hypothesize that constrained annealing of PLA—specifically, resin-based annealing via RTV silicone encapsulation—compared against granular salt improves dimensional stability and enables successful autoclave sterilization at 134 °C without structural deformation. To test this, we quantify absolute change (Δ, mm) and percentage change (%Δ relative to CAD nominal) across multiple geometric features and evaluate a small, complex, functional medical part (Easy Bone Collector shell). Our focus is on application-level dimensional outcomes rather than full thermal/mechanical spectroscopy, providing a practical, solvent-free protocol and comparative evidence to guide users toward sterilizable PLA components [5,23,24,25].

### 1.3. Context Within the State of the Art

The literature on improving PLA’s heat resistance and dimensional stability spans optimization of print parameters, annealing windows, and material strategies (e.g., blends and additives) [3,6,7,13,14,28,29,30,31]. These efforts collectively demonstrate that annealing can enhance crystallinity, heat deflection temperature, and mechanical robustness, while careful process tuning can reduce warpage and shrinkage. Broader advances in reinforced and architected structures—such as continuous-fiber reinforcement and non-planar toolpaths—offer additional routes to stiffness and thermal performance, but they do not directly address steam sterilization of neat PLA parts [32,33]. Against this backdrop, our work targets a practical, geometry-preserving constrained-anneal followed by a 134 °C steam cycle, quantified at the feature level and on a real device surrogate.

A concise synthesis of prior work (process parameters, annealing regimens, and sterilization outcomes) is provided in Table 1.

Upon annealing near/above Tg, PLA undergoes stress relaxation and chain interdiffusion at inter-bead interfaces, while cold crystallization increases ordering of the matrix. The combined effect reduces amorphous-phase mobility and raises the onset of softening/creep, thereby improving heat resistance. In our constrained setups, RTV encapsulation provides more uniform support during shrinkage associated with ordering, which aligns with the lower dimensional drift we measure after anneal and after the 134 °C steam cycle.

### 1.4. Originality and Contributions

As novelty, we deliver the first head-to-head comparison of salt embedding versus RTV-silicone (resin) encapsulation as constrained-annealing media for FDM-PLA, explicitly linked to a 134 °C steam cycle, on the same printing baseline and geometry set, with quantitative dimensional metrics (Δ, %Δ) reported for both generic features and a clinically relevant component.

Contributions:

1. We report absolute (Δ, mm) and relative (%Δ vs. CAD) dimensional changes pre-/post-anneal, evidencing the link between constrained annealing and post-sterilization dimensional fidelity.
2. We provide a bench-practical, solvent-free workflow (print → constrained anneal → sterilize) that complements prior work focused on materials development (blends, additives) or mechanical testing alone [3,13,14,28,29,30,31].
3. We supply actionable guidance on choosing the annealing medium (RTV vs. salt) for small, intricate PLA parts designed for steam sterilization.

Compared to prior work:

1. Earlier high-pressure studies show layer consolidation but are not bench-practicable and do not compare accessible constraint media [5].
2. Geometry retention after steam has been shown, but typically without a salt-vs-RTV comparison on the same platform and without Δ/%Δ tied to the steam cycle [10,12,21].
3. Demonstrations exist, yet no head-to-head salt-vs-RTV and no steam-linked dimensional metrics at 134 °C [23,24,25].
4. Useful context, but they do not resolve the practical choice of annealing medium for post-steam dimensional fidelity; our study fills this gap [3,13,14,28,29,30,31].

## 2. Materials and Methods

### 2.1. Materials

The filament used in this study was Ultrafuse^®^ PLA Tough (Forward AM (Heidelberg, Baden-Württemberg, Germany), BASF) [34], a biobased and impact-resistant PLA formulation designed for professional 3D printing applications. The physical, mechanical and thermal properties were provided in the extended technical data sheet and are presented in Supplementary Materials S1.

These properties confirm that Ultrafuse^®^ PLA Tough is a mechanically reinforced and thermally stable PLA formulation. Its compatibility with annealing and improved HDT/Vicat softening points post-treatment make it a suitable candidate for thermally processed and autoclave-sterilized 3D printed components.

In Table 2 are presented the materials we used in our research:

### 2.2. Standard Test Specimens

#### 2.2.1. Hollow Cylinders

**Figure 1:**
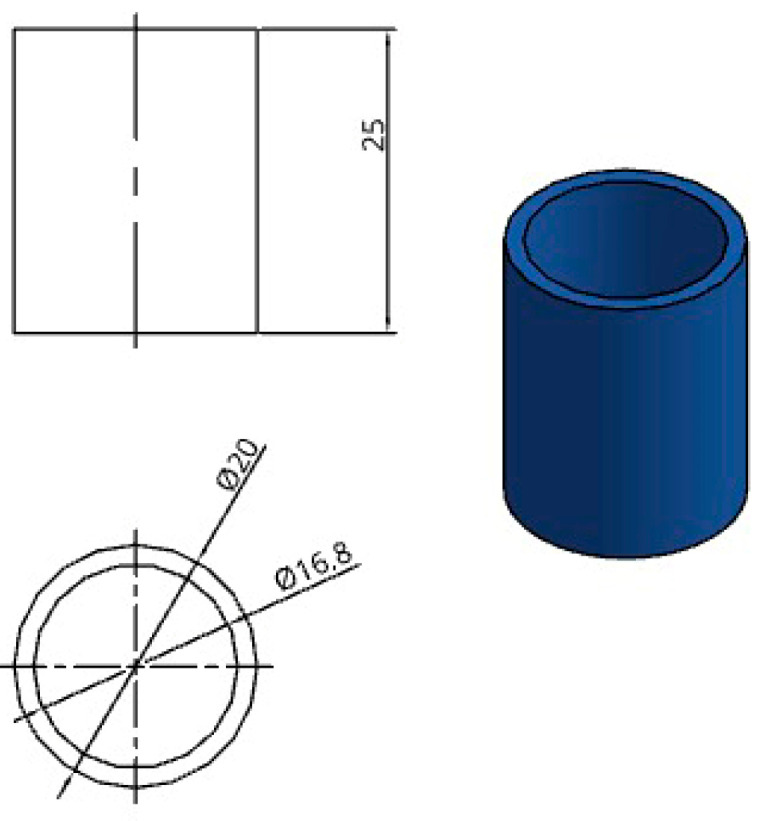
Dimensions and CAD model descriptions for hollow cylinders.

One of the geometries selected for evaluating thermal deformation and dimensional stability was a thin-walled hollow cylinder, as shown in Figure 1. The component’s dimensions are 16.8 mm for the inner diameter, 20 mm for the outer diameter, and 25 mm for the height.

The calibration specimen in Figure 1 was sized to be representative of the functional shell evaluated in the application study and to ensure robust printing, post-processing, and metrology. The inner and outer diameters (16.8 mm and 20 mm) give a wall thickness of 1.6 mm, matching the target shell thickness and yielding approximately four perimeters with a 0.4 mm nozzle, which reduces contour–infill interaction and improves repeatability during annealing. The 20 mm outer diameter is sufficient for accurate ID/OD gauging with calipers while enabling thermal equilibration and multiple replicates per build. A 25 mm height provides a representative number of stacked layers (≈100–125 at 0.20 mm layer height), making the part sensitive to Z-direction deformation yet compatible with embedding fixtures and sterilization packaging.

#### 2.2.2. Rectangular Bar

**Figure 2:**
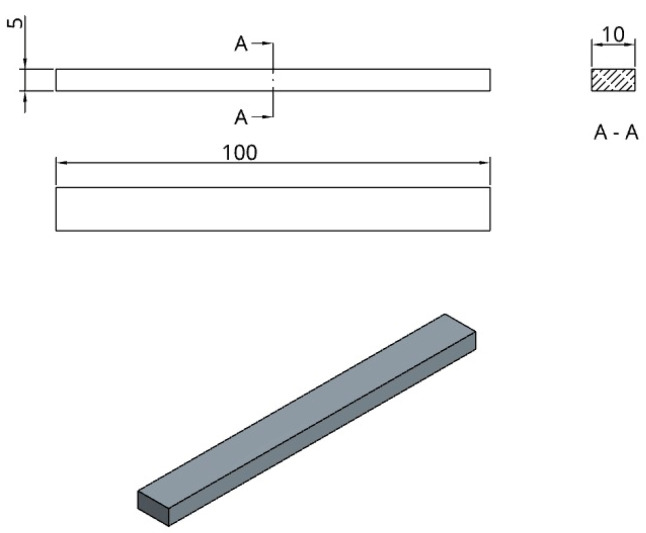
Dimensions and CAD model descriptions for rectangular bar.

The study evaluated longitudinal deformation, bending behavior, and thermal creep under the influence of heat and gravity using a rectangular bar specimen (Figure 2). The bar is 100 mm long, 10 mm by 5 mm in cross-section, and was printed horizontally with its longest axis pointing in the X direction of the printer.

#### 2.2.3. Curved Beam

**Figure 3:**
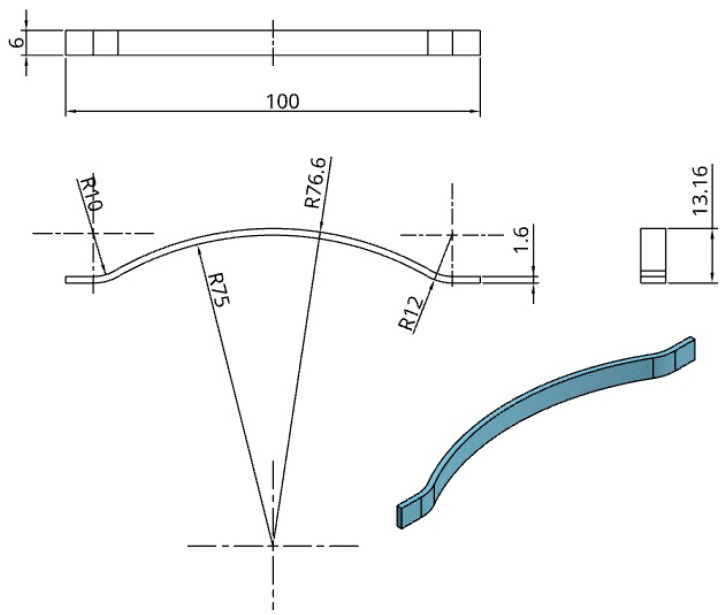
Dimensions and CAD model descriptions for curved beam.

To investigate the influence of complex surface curvature and bridge geometry on dimensional deformation during thermal post-processing, we included a curved beam specimen, as illustrated in Figure 3. The item has varied curvatures, a maximum height of 13.16 mm, and dimensions of 100 mm in length and 6 mm in breadth.

### 2.3. Functional Component Used to Validate Our Research: Easy Bone Collector Shell

As part of the applied section of this study, we selected a geometrically complex and small-scale functional component known as the Easy Bone Collector shell, used in intraoral clinical procedures and surgical setups.

**Figure 4:**
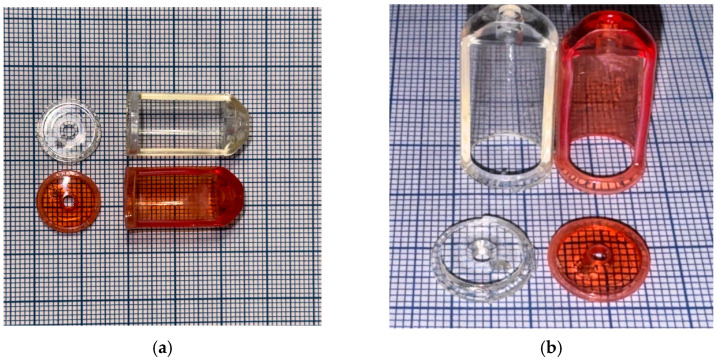
Original Easy Bone Collector Shell: The white one is before autoclaving, and the red one is after autoclaving: (**a**) Front view; (**b**) Top view.

The reason for selecting this shell is that the Easy Bone Collector originally comes with transparent ABS housing, which cannot withstand autoclaving, as it deforms at 121 °C to the extent that it can no longer be used, as you can see in Figure 4.

**Figure 5:**
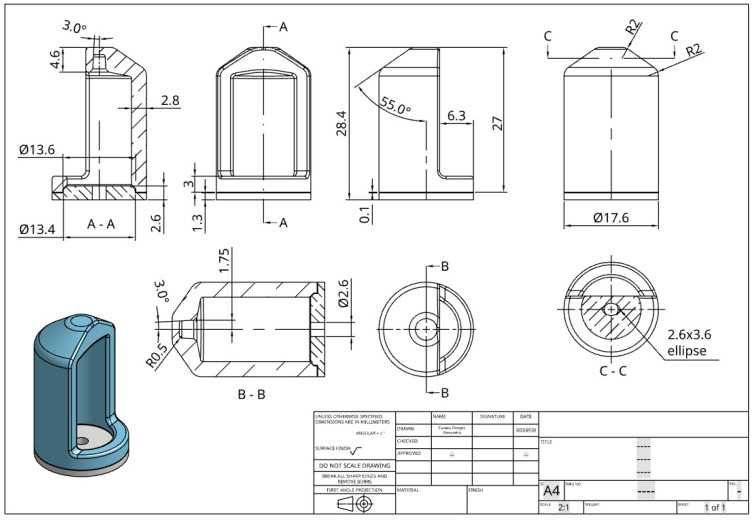
Dimensions and CAD model descriptions for Easy Bone Collector Shell.

The part, shown in Figure 5, is designed to interface with collection and stabilization systems for bone or tissue fragments during guided interventions. This shell has the role of limiting the height of the bone that is being cut, and it also has a protection role, by not allowing the medic to drive the cutter further than needed in the procedure. Another important function of the shell is to allow the cutter to rotate at high speeds, without any vibrations and without being deformed by the short time high temperatures and friction between the top and bottom of the shell and the cutter axel. The shell is composed of two parts that are being assembled with the cutter inside and sealed/glued together using epoxy resin that is heat resistant—ETN-T13-32/700, two parts, hardened with—M1142 one part.

Functional, anatomical, and handling restrictions were taken into consideration when modeling and sizing the component. It has a 17.6 mm diameter cylindrical outer body and a Ø13.4 mm internal cavity that fit typical insertion mounts. The total height reaches 28.4 mm, and the design includes a side cut-out of 6.3 mm, which facilitates lateral access during placement or removal in constrained working areas. The top surface is slanted at a 55° angle to facilitate ergonomic orientation and operation.

The following are important geometric features:

- Reduced localized stress concentrations by reinforced edges and transitions with rounded corners up to R2.0 mm;
- An elliptical slot (2.6 × 3.6 mm) and an internal alignment guide (Ø2.6 mm) made to precisely fit into reusable positioning frames;
- The component is especially relevant for evaluating dimensional stability during thermal processing and sterilization because of its thin wall sections, which are as low as 1.3 mm.

This design incorporates several difficult elements for thermal post-processing, such as compound surfaces, thin unsupported sections, and strict geometrical and dimensional tolerances. Confirming whether the recommended post-processing methods, such as autoclaving and salt/resin embedding, can preserve the mechanical and dimensional integrity of functional PLA parts meant for real biological applications is therefore a crucial test case.

### 2.4. Specimen Design and Classification

Four geometrically different specimens—a hollow cylinder, a rectangular bar, a curved beam, and a functioning radiography cap—were chosen in order to methodically assess the dimensional behavior and thermal stability of PLA components exposed to post-processing. From basic axial symmetry to intricate compound curves and functional interfaces, these examples were selected to highlight a variety of structural features commonly observed in practical applications.

Table 3 summarizes the particular difficulties that each specimen poses with regard to surface exposure, crucial geometric transitions, and wall thickness. For example, the curved beam magnifies the effects of thermal stress because of its bridge shape and unsupported arch; the rectangular bar is perfect for evaluating bending and creep under its own weight; the hollow cylinder allows for radial deformation and ovalization analysis; and the Easy Bone Collector shell replicates a clinically relevant use-case involving precise fit, thin features, and sterilization compatibility. When combined, these geometries offer a thorough experimental framework for assessing the effects of solid-media thermal post-processing on warping tendencies, dimension preservation, and overall geometric fidelity in a variety of part types.

### 2.5. Methodology

**Figure 6:**
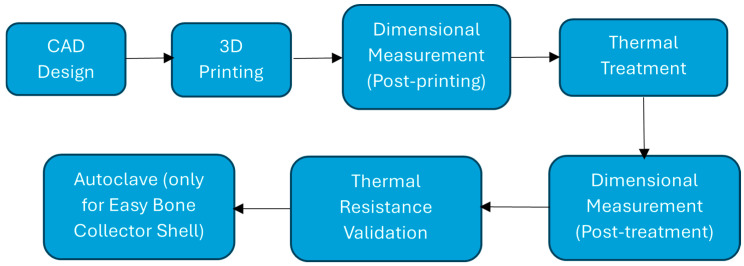
Shows the experimental workflow from digital design to final thermal validation. This structured process ensured consistent evaluation across all specimen geometries and embedding conditions.

The experimental procedure consisted of seven distinct stages, as illustrated in Figure 6. Each step was designed to evaluate the effects of thermal post-processing on the dimensional stability and heat resistance of 3D printed PLA components.

1. 1. CAD Design

All test specimens and the functional component (Easy Bone Collector shell) were made using parametric 3D modeling software to ensure geometry accuracy and repeatability.

2. 3D Printing

The parts were made using fused deposition modeling (FDM) technology and Ultrafuse^®^ PLA Tough filament. The manufacturer’s technical data sheet instructions were followed while setting the print parameters.

3. Dimensional Measurement (Post-printing)

After each component was fabricated, its measurements were taken with calipers and digital microscopes. These provide a place to start for evaluating deformation and shrinkage.

4. Thermal Treatment

The components were subjected to thermal post-processing in compliance with the annealing protocol recommended by the filament supplier. The geometry was supported while it was heated by embedding it in RTV silicone resin or fine NaCl powder.

5. Dimensional Measurement (Post-treatment)

Dimensions were re-measured following the heat treatment in order to measure distortion, shrinkage, and any loss of geometric integrity.

6. Thermal Resistance Validation

To simulate sterilization scenarios, parts were subjected to autoclave cycles. Dimensional stability and visual integrity were evaluated after each cycle to determine the suitability of each embedding method.

7. Autoclave (only for Easy Bone Collector)

Only the Easy Bone Collector with the 3D printed shell was autoclave sterilized once the thermal resistance tests were completed on the standard specimens (cylindrical, rectangular bar, and curved beam). Because of its intricate geometry, which includes thin walls, undercuts, and tight tolerances, as well as its practical relevance in clinical settings, this component was chosen for sterilization testing. Since the standard specimens demonstrated structural integrity at temperatures above 170 °C without deformation, it is reasonable to believe that similar designs can withstand autoclave conditions. The sterilization test only looked at the most challenging and application-relevant part of the process in order to validate it under real-world conditions.

#### 2.5.1. Three-Dimensional Printing Parameters and Orientation

All specimens were fabricated using a Fused Deposition Modeling (FDM) printer, specifically the Sovol SV06, equipped with a 0.4 mm nozzle. The printing material was Ultrafuse^®^ PLA Tough, selected for its impact resistance and enhanced thermal properties. The following printing parameters, Table 4, were consistently applied throughout the study:

A single nominal speed of 60 mm/s was used for all generated toolpaths. Because the geometries are thin and small, the parts were printed with perimeters only (no bridges/supports). This speed was chosen after pilot prints to maximize layer uniformity and dimensional consistency on small features; faster settings increased variance without measurable benefit for our endpoint (dimensional accuracy).

Slicing was performed in PrusaSlicer 2.9.2, with all models arranged in a single print job to minimize build-to-build variability. Orientations were chosen to excite distinct distortion modes: the hollow cylinder was printed upright to probe radial shrinkage along the build axis; the rectangular bar was printed flat (100 × 10 mm face) to be sensitive to gravity sag/thermal creep; the curved beam was printed arch-up to challenge unsupported curvature. The Easy Bone Collector shell used perimeter-dominant toolpaths with minimal paint-on supports to preserve surface tolerances. Full layouts, support strategy, and time/material estimates are provided in Supplementary Materials S2.

#### 2.5.2. Embedding Media and Thermal Treatment Protocol

Each specimen was entirely embedded in one of the two media types and placed inside a laboratory oven with programmable thermal control.

The thermal treatment was carried out in the following staged protocol, Table 5:

This temperature profile was derived from the manufacturer’s technical data sheet for Ultrafuse^®^ PLA Tough and was selected to remain below the material’s degradation point while sufficiently exceeding its glass transition temperature (~60–65 °C). According to the literature, implementing a staged ramp reduces thermal shock and facilitates progressive stress relaxation [11,12]; the subsequent isothermal dwell promotes chain mobility and rearrangement, which has been associated with higher crystalline order [3,6,7,14,28,30].

**Figure 7:**
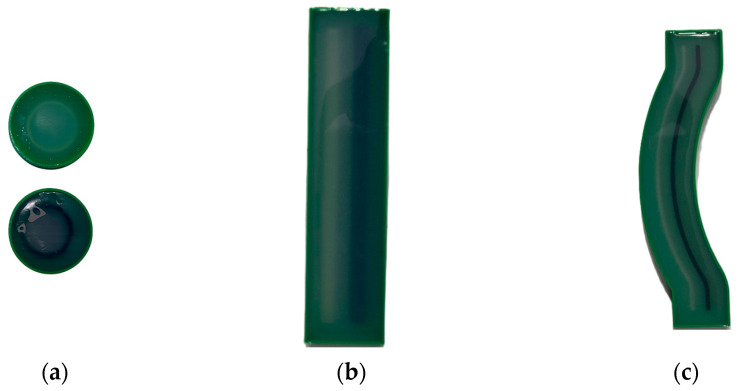
Specimens encapsulated in RTV silicone resin (SVSIL-P25) prior to thermal annealing. The resin molds provided a fully constrained environment for deformation-sensitive geometries: (**a**) hollow cylinders, (**b**) rectangular bar, (**c**) curved beam.

The samples were prepared in RTV silicone molds, as seen in Figure 7, where the fluid resin was poured around the specimens and left to dry completely before being placed in the oven. The resin molds were appropriate for delicate or intricate parts because they offered consistent surface contact, minimal mechanical constraint, and chemical inertness.

**Figure 8:**
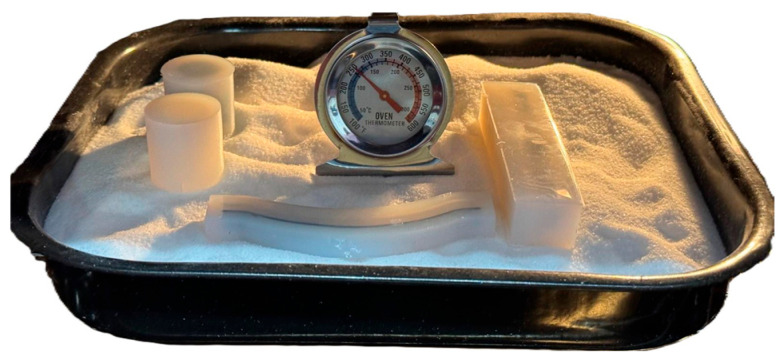
Final experimental setup for the thermal annealing process: PLA specimens encapsulated in RTV silicone molds and positioned in a salt-filled tray, with an analog thermometer used to monitor the internal temperature during the heat cycle.

The use of granular salt embedding, on the other hand, is demonstrated in Figure 8, where the components were arranged on a heat-resistant tray that was filled with NaCl. The salt medium prevented deformation during heating and cooling, provided equal heat distribution, and provided stiff physical support. To keep an eye on and verify the internal temperature profile separately from the oven’s digital control, an external oven thermometer was positioned next to the components.

To guarantee dimension stability and reduce mechanical distortion during annealing, a regulated setup was created for the thermal post-processing step. The image above shows the PLA specimens in their final configuration, ready for oven treatment. The printed items were coated with a uniform layer of fine sodium chloride (NaCl), which served as both a heat transfer medium and a basis for mechanical support. In order to provide structural support and shape preservation throughout the heat cycle, each specimen was individually enclosed in a rigid RTV silicon mold (SVSIL-P25) on top of this salt bed.

This widely used heat treatment technique, which entails simultaneously placing silicone-supported parts and salt in the same oven, allows all PLA samples to be annealed at the same setting. While the cured silicone molds limit deformation and maintain the original geometry of even the most delicate or thin-walled structures, the salt disperses heat uniformly and avoids localized overheating.

#### 2.5.3. Dimensional Measurement

**Figure 9:**
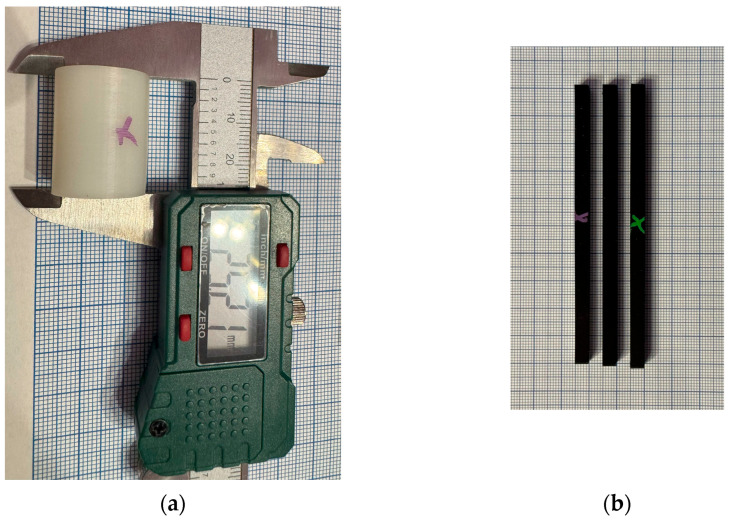
Measurement setup used for evaluating dimensional accuracy. (**a**) Digital caliper; (**b**) millimeter grid paper used to assess specimen length, width, and thickness.

To facilitate visual and planar dimensional measurements, particularly when assessing distortion or warping along the specimen’s length, a millimeter-grid paper was used as a reference background (Figure 9). After post-processing, layer consistency, warping, and material surface integrity may be qualitatively observed thanks to a digital optical camera system in regulated lighting for surface quality and texture evaluation.

Dimensional measurements (Δ in mm; %Δ vs. CAD) were performed after printing and after constrained annealing (salt or RTV). For the steam-sterilization step (134 °C), we conducted a functional fit check on the application component (EBC) rather than caliper-based measurements on all samples; quantitative post-steam metrology is planned as follow-up work. The formulas for the measurement are presented in Table 6.

Replicates: n = 6 per geometry and condition (four specimens from the same build annealed together + two confirmatory specimen). For each geometry, we used n = 3 replicates per material.

#### 2.5.4. Autoclave Sterilization Procedure

Steam sterilization at 134 °C for 60 min (medical pouch). Dimensional checks confirmed geometry retention and fit for the EBC shell post-cycle.

To replicate actual use circumstances, components were enclosed in sterilizing pouches of the highest caliber for medical use. Preheating, steam exposure at the predetermined temperature and pressure, and a drying step to remove any remaining moisture were all part of the procedure. Test specimens were subjected to both thermal and hydrolytic stress conditions that were typical of clinical sterilizing settings according to this methodology.

#### 2.5.5. Evaluation Criteria

A set of quantitative and qualitative evaluation criteria was created in order to assess the effectiveness of thermal post-processing and sterilization. These criteria focused on geometric fidelity, surface quality, resistance to post-treatment deformation, and dimension retention in order to give a comprehensive understanding of how each treatment condition affected the PLA specimens’ mechanical and morphological stability.

Dimensional retention was measured by comparing key measurements (length, width, wall thickness, and internal dimensions) before and after annealing and autoclaving. In dental and medical applications where precise alignment is necessary, changes larger than ±0.2 mm were deemed significant. Internal features and fit-dependent locations, including the Easy Bone Collector’s elliptical guide groove and the hollow cylinders’ wall thickness, received special attention.

Geometric fidelity was evaluated by visual inspection and overlay comparison on millimeter paper grids, searching for any asymmetrical distortions such as warping or drooping. The degree to which each embedding medium maintained form throughout temperature cycles was assessed using geometries that were particularly sensitive to deformation, such as those having curves, cutouts, or unsupported spans (such as the curved beam and radio-graphic cap).

To detect delamination, variations in surface roughness, or surface layer displacement, surface quality was assessed by optical photography under uniform illumination. The surface finish of pristine and annealed parts was assessed qualitatively by standardized macro-photography. Imaging was performed on a fixed stand with diffuse, oblique illumination (~45°) to highlight layer lines and defects; camera settings and working distance were kept constant across conditions. No contact or optical profilometry was performed; therefore, no Ra/Rq values are reported.

## 3. Results and Discussion

Dimensional outcomes are reported for the post-anneal state (Δ in mm; %Δ vs. CAD). The standard specimens (cylinder, bar, curved beam) were exposed to elevated temperatures in a dry oven (100–175 °C) to simulate the thermal load of sterilization, but we did not acquire caliper-based post-steam (134 °C) measurements for these samples. Steam autoclaving at 134 °C was performed only on the functional Easy Bone Collector (EBC) shell, for which we confirmed post-steam fit/function; quantitative post-steam metrology across samples is planned as follow-up work.

For each geometry and thermal condition (as-printed, salt-annealed, RTV-annealed), we used n = 6 specimens: four printed in the same build and annealed simultaneously, plus two additional confirmatory specimens manufactured and processed under the same profile. For each geometry, we used n = 3 replicates per material. However, for clarity and visual relevance, only two representative specimens per group are presented in the paper research. The data analysis presented in the article is based on the average values obtained from all six samples, ensuring statistical reliability while maintaining a concise and readable format.

Across all replicate sets (n = 6 per geometry), the directional trend was consistent (salt → larger contraction/ovalization; RTV → minimized drift), while the absolute magnitude of Δ and %Δ remained geometry-dependent.

**Figure 10:**
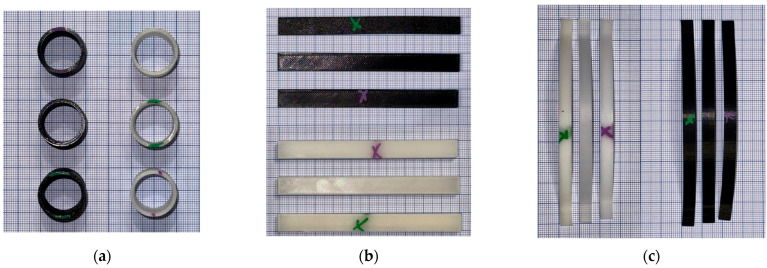
PLA specimens after thermal post-processing and prior to thermal resistance testing. (**a**) Cylindrical samples (top view), (**b**) rectangular bar specimens, (**c**) curved beam specimens. In each case, unmarked = untreated, green “X” = annealed in silicone resin, purple “X” = annealed in salt.

Figure 10 presents the three PLA specimen types after thermal post-processing and prior to heat resistance testing. Each set includes one untreated sample (unmarked), one sample annealed in RTV silicone resin (green “X”), and one annealed in salt (purple “X”). These three geometries were selected in order to evaluate the impact of shape complexity on annealing effectiveness: curved beams (c), rectangular bars (b), and cylindrical rings (a). All of the specimens at this point maintained their original geometry, making it possible to evaluate the deformation in future high-temperature exposures with clarity.

**Figure 11:**
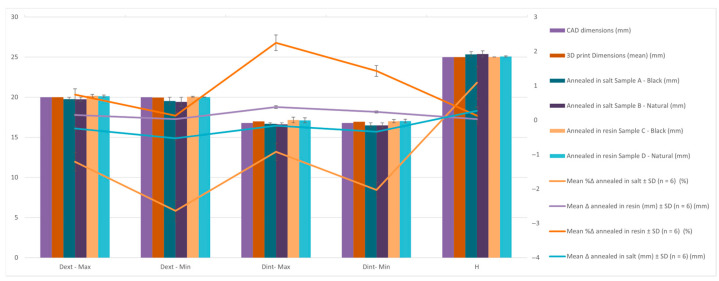
Graphic representation of the dimensional evaluation of hollow cylinder. Dimensional deviation relative to CAD across processing stages (as-printed, salt-annealed, RTV-annealed): mean ± SD, Δ is reported in mm and %Δ is shown on the secondary axis. Per replicate values and calculation details are provided in the Supplementary Materials.

Table 7 and Figure 11 present the comparative dimensional data for Hollow Cylinder specimens in their CAD design, as-printed state, and after annealing in salt and resin. Six post-annealing samples are analyzed (A–F), covering both black and natural PLA Tough materials, under two distinct annealing media. Mean Δ, SD Δ, Mean %Δ and SD %Δ are graphically presented in the figure below and the values can be found for all samples and sample types in the Supplementary Materials S3.

**Figure 12:**
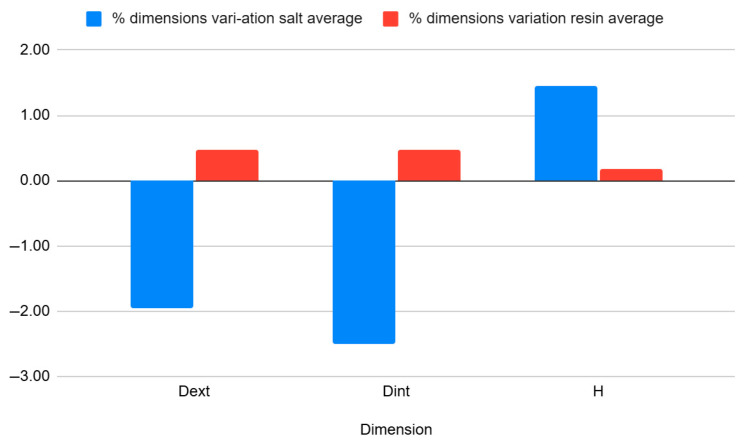
Graphic representation of the percentual variation in dimensions average of hollow cylinder specimens after annealing in salt and resin.

Table 8 and Figure 12 shows the average percentage variation in the dimensions of hollow cylinder specimens after heat treatment by annealing in salt and in resin. It can be observed that salt treatment resulted in a reduction of the outer diameter (−1.95%) and inner diameter (−2.50%), while the height increased slightly (+1.46%). In contrast, resin treatment had a much smaller effect on the dimensions, with positive variations of approximately 0.47% for the diameters and 0.18% for the height, indicating better dimensional stability.

Findings:

- Outer Diameter (Dext):
  ○ The CAD outer diameter was Ø20.00 mm.
  ○ After printing, it slightly increased to Ø20.03 mm—typical for PLA expansion.
  ○ Salt-annealed samples show notable contraction (as low as Ø19.43 mm in Sample B), indicating radial shrinkage and ovalization.
  ○ In contrast, samples C and D that were resin-annealed showed little deformation and maintained outside diameters that were almost nominal (Ø20.18 mm and Ø20.14 mm).
- Inner Diameter (Dint):
  ○ CAD value was Ø16.80 mm; printed part slightly expanded to Ø17.02 mm.
  ○ Salt-treated samples shrank internally to Ø16.68 and Ø16.62 mm (Max), and Ø16.50 mm (Min), a potential result of material softening and collapse of unsupported areas.
  ○ Resin-annealed parts demonstrated improved cavity preservation and once again maintained higher dimensional precision (Max: Ø17.16 mm, Min: Ø17.01 mm).
- Height (H):
  ○ CAD and 3D printed height was 25 mm.
  ○ After salt treatment, specimens experienced vertical expansion (up to 25.39 mm, Sample B), suggesting unrestrained material flow under thermal stress.
  ○ Resin-embedded samples remained nearly constant (25.01 mm to 25.08 mm), indicating better control in the vertical direction.

Conclusion:

The findings show that when cylindrical PLA components are thermally annealed, resin embedding effectively maintains dimensions. While salt embedding results in considerable deformation, particularly shrinkage in both axial and radial directions, resin molds maintain geometry with exceptional fidelity regardless of material color. This supports the notion that resin-based thermal post-processing is more appropriate for parts with internal cavities or circular cross-sections, where tolerances are essential.

**Figure 13:**
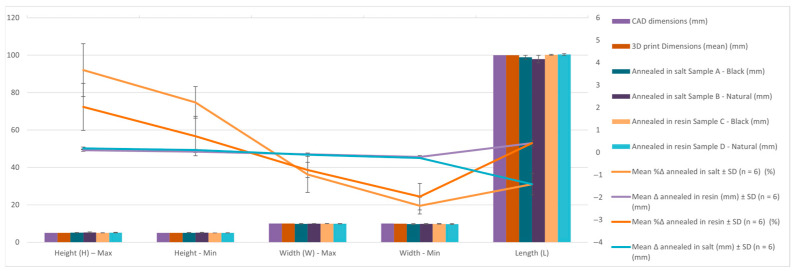
Graphic representation of the dimensional evaluation of rectangular bar specimens. Dimensional deviation relative to CAD across processing stages (as-printed, salt-annealed, RTV-annealed): mean ± SD, Δ is reported in mm and %Δ is shown on the secondary axis. Per replicate values and calculation details are provided in the Supplementary Materials.

Table 9 and Figure 13 presents measurements of the rectangular bar specimen, with a nominal height of 5 mm, width of 10 mm, and total length of 100 mm. Measurements were collected after 3D printing and after annealing in salt and resin, for both black and natural PLA Tough variants.

**Figure 14:**
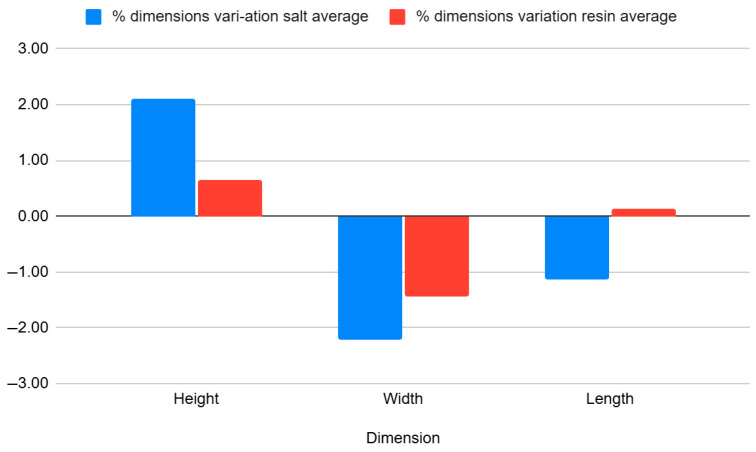
Graphic representation of the percentual variation in dimensions average of rectangular bar specimens after annealing in salt and resin.

Table 10 and Figure 14 show the average percentage variation in the dimensions of rectangular bar specimens after heat treatment by annealing in salt and in resin. It can be observed that salt treatment resulted in an increase in height (+2.10%) and a reduction in width (−2.22%) and length (−1.14%). In contrast, resin treatment caused smaller dimensional changes, with a slight increase in height (+0.64%), and decreases in width (−1.43%) and length (−0.12%), indicating better dimensional stability compared to salt treatment.

Findings:

- After salt annealing, specimens showed significant contraction, especially in length (L) where values dropped to 98.85 mm and 98.02 mm—a deviation of nearly 2% from the CAD model.
- Height and width dimensions also increased slightly (e.g., H-max: 5.23 mm), indicating thermal swelling or layer relaxation.
- Specimens annealed in resin, on the other hand, showed more balanced height and width profiles and retained CAD-aligned dimensions (e.g., L = 100.25–100.4 mm).

Conclusion:

While salt annealing resulted in warping and axial contraction, resin embedding showed superiority in maintaining global geometry and length. This was probably driven by uneven constraint and inadequate temperature control for thin sections.

**Figure 15:**
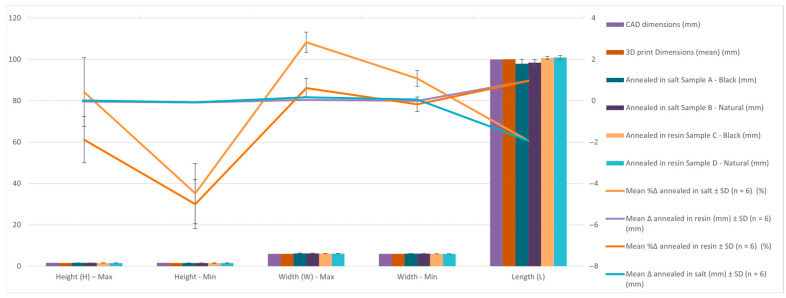
Graphic representation of the dimensional evaluation of curved beam specimens. Dimensional deviation relative to CAD across processing stages (as-printed, salt-annealed, RTV-annealed): mean ± SD, Δ is reported in mm and %Δ is shown on the secondary axis. Per replicate values and calculation details are provided in the Supplementary Materials.

Table 11 and Figure 15 summarize results for the curved beam geometry, which features thin walls (1.6 mm) and a nominal arc length of 100 mm. Due to its bridge structure, this geometry is highly sensitive to thermal deformation.

**Figure 16:**
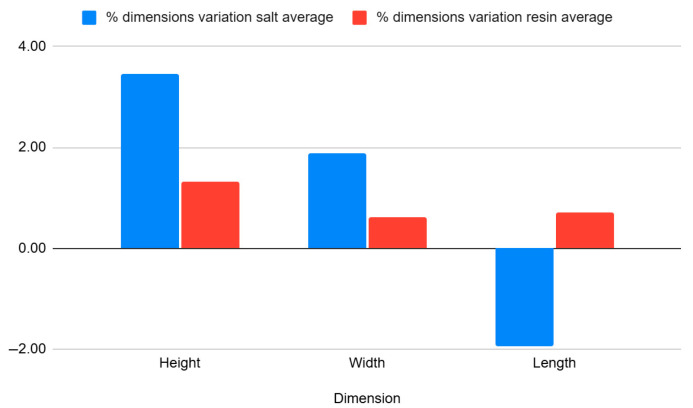
Graphic representation of the percentual variation in dimensions average of curved beam specimens after annealing in salt and resin.

Table 12 and Figure 16 show the average percentage variation in the dimensions of curved beam specimens after heat treatment by annealing in salt and in resin. It can be observed that salt treatment resulted in a significant increase in height (+3.53%) and width (+1.88%), while the length decreased (−2.01%). In contrast, resin treatment produced smaller dimensional changes, with increases in height (+1.36%), width (+0.63%), and length (+0.63%), indicating better overall dimensional stability compared to salt treatment.

Findings:

- After annealing in salt, the parts shrank significantly along the arc length (e.g., L = 97.91 mm and 98.46 mm), and exhibited higher variation in height (H-max: 1.6 mm; H-min: 1.5 mm).
- In contrast, specimens that were resin-annealed maintained their size and form well, with constant height values (1.52–1.56 mm) and arc lengths of 100.75 mm and 100.92 mm.

Conclusion:

Resin embedding provides efficient dimensional stability for designs with intricate, unsupported curves, avoiding torsional warp and curvature collapse. Because of their unequal restriction and development of thermal stress, salt-embedded samples are not appropriate for such applications.

**Figure 17:**
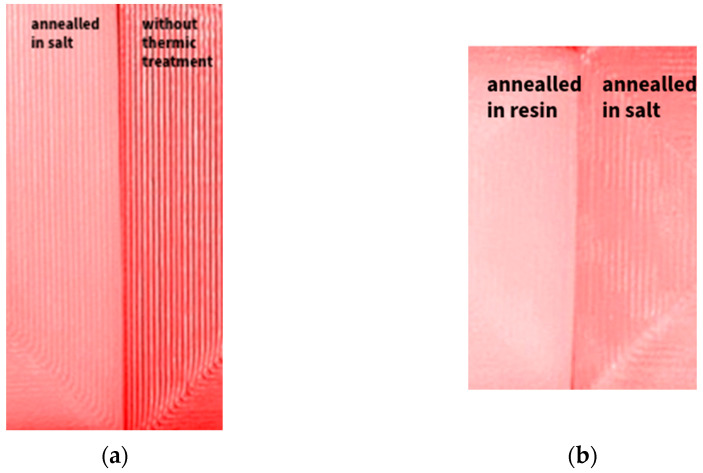
Visual inspection (macro-photography) of pristine vs. annealed surfaces under standardized lighting; images acquired with constant settings. (**a**) Surface detail comparison between a PLA part annealed in salt and an untreated (non-annealed) part, (**b**) comparison between surface finishes of PLA annealed in resin and in salt. Annealed samples exhibit smoother top layers with improved layer cohesion compared to the untreated sample.

The apparent variations in surface quality caused by different thermal post-processing techniques used on PLA specimens are depicted in Figure 17. Surface inspection was visual qualitative; no profilometry was conducted. Our primary endpoint was dimensional stability under 134 °C steam sterilization. A distinct contrast between a part that has been annealed in salt and one that has not undergone any thermal treatment can be seen in Figure 17a. Particularly in the curved areas where complete fusion is not attained, the untreated sample exhibits noticeable distance between print layers and prominent layer lines. On the other hand, the annealed portion exhibits better top layer cohesiveness, less pronounced individual layers, and increased surface continuity. Parts annealed in salt and RTV silicone resin are contrasted in Figure 17b. The resin-annealed sample exhibits the most polished finish, with better interlayer bonding and less surface roughness, even though both treatments provide a surface that is noticeably smoother than the untreated control. These findings demonstrate that heat treatment greatly enhances the functional and visual surface quality of 3D-printed PLA parts in addition to improving thermal and dimension stability.

The sample specimens were tested in the oven after annealing to determine the maximum heat that each specimen type can handle without any visible deformation. The hollow cylinders were tested in standing position and laid on a side, the curved beam were set on the small flat ends, and the rectangular bars were set with the ends on taller surfaces to allow them to deform under their own weight. Each sample type had one printed crude part, one annealed in salt (marked with purple) and another in resin (marked with green).

**Figure 18:**
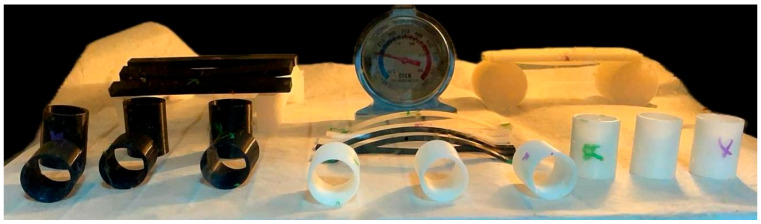
Full set of PLA specimens after thermal exposure at 100 °C. From left to right: rectangular bars, curved beam springs, and hollow cylindrical samples, all positioned in an un-preheated oven at controlled temperature.

As shown in Figure 18, the results confirmed the expected behavior of untreated PLA parts. The crude, non-annealed specimens began to deform at relatively low temperatures, with the curved beam geometry exhibiting visible warping from approximately 70 °C, while rectangular bars and hollow cylindrical samples resisted deformation until nearing 100 °C. These differences are attributed to geometric rigidity, but in all cases, untreated PLA proved unsuitable for applications involving elevated temperatures.

**Figure 19:**
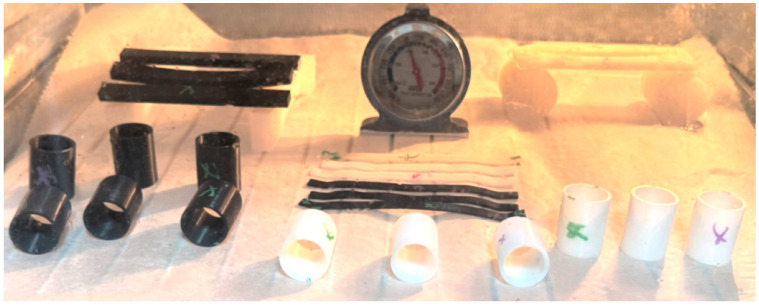
Full set of PLA specimens after thermal exposure at 150 °C. From left to right: rectangular bars, curved beam springs, and hollow cylindrical samples, all positioned in a preheated oven at controlled temperature.

Figure 19 shows the complete collection of printed PLA geometries arranged for thermal exposure at 150 °C. Each geometry—rectangular bars, curved beams, and hollow cylinders—was represented by multiple specimens with different thermal treatments: untreated, annealed in salt, and annealed in RTV resin (marked accordingly). This setup allowed simultaneous observation of deformation thresholds under identical conditions. The thermometer positioned centrally validates the internal oven temperature during testing, ensuring accurate assessment of thermal behavior across treatments and geometries.

**Figure 20:**
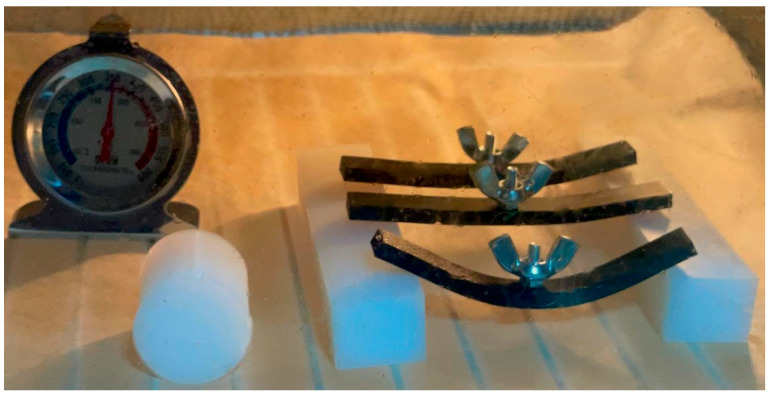
Rectangular bar PLA specimens mounted in a test rig for thermal deformation evaluation.

The annealed parts both salt and resin supported were able to handle temperatures up to 130 °C for the curved beam and up to 175 °C for the rectangular bar where we added an external load of 6.2 g in the middle of each sample, Figure 20.

From these results we can conclude that the curved beam type annealed prints (thin prints with long unsupported bridges) may only be autoclaved at low temperatures for short times (121 °C for 15 min) and bar type or cylindrical parts annealed may be autoclaved at the top temperatures and for any time as they will not deform up to 175 °C.

Easy Bone Collector shell assembled as a product—was subjected to autoclave cycle after undergoing thermal post-processing of each part in resin. After the autoclave process was performed the product was inspected visually and it was used on bone-like materials to validate that the cutter will work properly even on dense bones structures. All the samples performed as expected, with successful cuts at different turbine speeds and all of the tests showed that the product could be successfully used on any human subject after autoclave at 134 °C and the product can safely be autoclaved again after each use.

**Figure 21:**
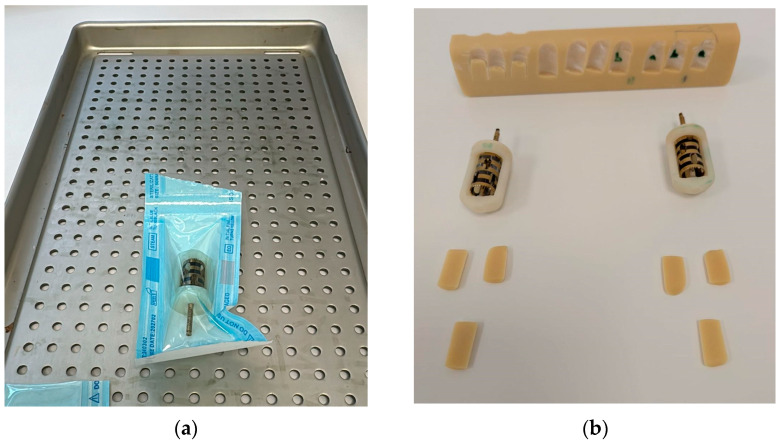
Final evaluation of the Easy Bone Collector shell: (**a**) component sealed in a sterilization pouch prior to autoclave sterilization; (**b**) post-sterilization assembly test and visual inspection of the Easy Bone Collector system, confirming part stability and compatibility with all subcomponents.

The final validation step involved testing the sterilization compatibility of the Easy Bone Collector shell, a medical device component with a complex geometry. As shown in Figure 21a, the part was sealed in a sterilization pouch and subjected to an autoclave cycle at 134 °C. After sterilization, the component was removed and visually inspected.

Figure 21b shows the post-sterilization assembly with all subcomponents fitted into the sterilized shell. The outcomes show the method’s practicality for medical-grade functional components by confirming that the annealed PLA part retained full mechanical compatibility and dimensional precision.

Annealing of PLA near/above enables chain mobility and stress relaxation and, as reported in prior studies, can promote ordering/crystallinity. Increased ordering is associated with enhanced resistance to softening and shape distortion at elevated temperatures. In our study we did not measure crystallinity; instead, we quantify the application-level outcome—dimensional stability under a 134 °C steam-sterilization cycle. The reduction in dimensional change (Δ, %Δ) observed after constrained annealing (salt/RTV-resin) is consistent with this literature-based rationale.

This study prioritized application-level dimensional metrics (Δ, %Δ) over microstructural spectroscopy. We did not quantify crystallinity by DSC nor inter-bead morphology by SEM; instead, we infer the mechanism from literature and vendor thermal data for the same PLA grade and from the observed geometry retention.

## 4. Conclusions

This study presents a practical approach to constrained annealing aimed at maintaining the geometry of FDM-PLA components during a 134 °C steam sterilization cycle. Conducted with six replicates for each geometry and condition, the experiments revealed that RTV-embedded annealing consistently resulted in the least amount of dimensional drift post-annealing and steam treatment, whereas the salt immersion method led to greater contraction and ovalization.

Specifically, the Easy Bone Collector component demonstrated preserved fit and functionality after 60 min at 134 °C when subjected to pre-annealing, in contrast to as-printed PLA, which exhibited deformation during the sterilization process. The primary metrics for evaluating success were quantitative measurements of geometry retention (Δ in mm; %Δ vs. CAD), with improvements showing consistent directional trends and effect sizes that depended on specific geometries.

The overall workflow—transitioning from printing to RTV-constrained annealing and then to steam sterilization—proves to be solvent-free and cost-effective. A significant advantage of this method is that the RTV resin, in its liquid state, allows for conformal wetting and complete encapsulation of the printed part during pre-curing. This technique effectively fills gaps and uniformly supports intricate features, undercuts, and thin-wall structures. Post-curing, the resulting flexible, non-adherent mold simplifies the demolding process and minimizes voids, as well as stress concentrations, compared to traditional granular media approaches.

In terms of novelty, this “mold-in-resin” constrained annealing method is rigorously compared to the salt technique that uses the same initial printing parameters, specifically linking it to a 134 °C steam cycle. This offers valuable insights for the production of sterilizable, dimensionally stable PLA parts, while also aiming to mitigate scrap and reduce reliance on single-use materials.

The limitations of this research primarily involve maintaining dimensional fidelity as the principal outcome. Future studies will explore comprehensive quantitative post-steam measurements (Δ/%Δ) across various geometries and will seek to establish correlations with DSC, SEM, HDT, TMA, and DMA analyses.

## Figures and Tables

**Table 1:** Representative studies on annealing and sterilization of FDM-PLA (condensed).

Ref	Focus	Material/Specimen	Print Setup (Key)	Annealing/Conditioning	Sterilization (Method/T)	Key Outcome	Relevance to This Work
[1]	Pressure-assisted heat	PLA test parts	FDM (var. params)	Pressure/temperature ≤ Tg (custom autoclave)	Steam, high pressure (custom)	↑ layer consolidation and strength; negligible warping under controlled pressure	Motivates constrained/pressure annealing
[5]	Anneal + sterilize model	PLA surgical retractor	FDM; geometry optimized	Pre-annealed	Steam autoclave 134 °C	Lower distortion; higher retained strength vs. non-optimized PLA	Direct link pre-anneal → autoclave survivability
[6]	Print params × anneal	PLA/PETG tensile bars	Layer height 0.1–0.3 mm	60–100 °C, 30–90 min	n/a	Layer height dominates tensile and dimensional outcomes	Guides selection of anneal/print parameters
[7]	Annealing optimization	PLA coupons	FDM	~90 °C × ~120 min (opt.)	n/a	Statistically significant ↑ properties; temperature dominant	Defines practicable anneal window
[10]	Sterilization across materials	PLA and others	n/a	n/a	Steam 121 °C (~2–3 bar)	Measured pre/post dimensional shifts by material/infill	Shows sterilization-induced drift; motivates pre-anneal control
[11]	‘Lazy’ annealing	PLA specimens	MEX/FFF	Passive constrained anneal	n/a	Warping reduced; properties ↑ up to ~14%	Supports physical constraint during anneal
[12]	Mold-constrained anneal	Thermoplastic polymers	FDM	Annealing in molds (NaCl/gypsum-like)	n/a	Maintained dimensions; improved properties via constraint	Directly relevant constrained-media approach
[13]	Co-optimization	PLA parts	FFF; RSM design	Optimized anneal + print	n/a	↑ dimensional accuracy and strength simultaneously	Process + anneal co-optimization path
[14]	Blend + anneal	PLA/PHB coupons	FDM	Annealing; PHB blending	n/a	↑ crystallinity/strength and thermal resistance; ↓ distortion	Alternative/complementary route to stability
[22]	Application-level sockets	Tough PLA socket parts	FDM	Oven annealing (var. media tested)	n/a	Deformation reduced to ~2% (sand medium); ↑ strength	Practical constrained medium improves accuracy
[23]	Sterilization effects	PLA specimens	FDM; varied infill/layer	n/a	Steam 121–134 °C	Print parameters drive dimensional/mech. change; pre-anneal recommended	Need to control print/anneal for stability
[24]	Clinical guides	PLA/HTPLA/nylon guides	FDM; optimized	Annealed HTPLA	Steam; multiple cycles	PLA/HTPLA retained geometry; nylon swelled	Crystalline (annealed) PLA suitable for guides
[25]	Clinical sterilization	PLA biomodels	FDM; internal grades	n/a	Steam 134 °C (clinical)	Minor deformation with optimal config; sterilization effective	Feasibility of 134 °C with right settings
[26]	Autoclavable devices	PLA bioreactor vessels	3D printed design	Geometry/process-enabled	Steam autoclave (biotech)	Autoclavable, biocompatible PLA vessels achieved	Application-level autoclave example
[27]	Sterilization review	3D-printed materials	n/a	n/a	Multiple methods	Comparative effects of sterilization on 3D-printed materials	Contextualizes steam vs. other methods

Note: Not all studies performed steam sterilization; when absent, we report annealing/processing insights relevant to dimensional stability and heat resistance. (HTPLA = high-temperature PLA; n/a = not applicable).

**Table 2:** Bill of materials and equipment.

Item	Model/Notes
FDM printer	Sovol SV06 (Sovol, Shenzhen, China); 0.4 mm nozzle
Filament	Ultrafuse^®^ PLA Tough (Forward AM/BASF)
Slicer	PrusaSlicer 2.9.2
Embedding medium #1	Extra-fine NaCl (full embedding)
Embedding medium #2	RTV silicone resin SVSIL-P25 (DEVE SRL, Oradea, Romania) (two-part; room-temperature cure)
Programmable oven	Laboratory oven with ramp/hold capability
Autoclave	Steam sterilizer, 134 °C cycle
Measurement tools	Digital calipers; millimeter grid; macro photo rig

**Table 3:** Comparative geometric specimens table.

Specimen Type	Main Dimensions (mm)	Wall Thickness (mm)	Critical Geometry Features	Thermal Deformation Sensitivity	Analysis Purpose
Hollow Cylinder	Ø20 × Ø16.8 × 25	1.6	Thin cylindrical wall	Ovalization, shrinkage	Radial and height
Rectangular Bar	100 × 10 × 5	5.0	Long, slender beam	Bending under own weight	Deflection and creep
Curved Beam	100 × 13.16 × 6	1.6 (at ends)	Multiple radii, symmetric unsupported arc	Warping, twist, symmetry, bending under own weight	Arc deformation and recovery
Easy Bone Collector shell	Ø17.6 × 28.4 × 13.4	varied (min 1.3)	Cut-outs, angles, internal slots	Dimensional drift, warping	Application-specific fit precision

**Table 4:** Full printer/slicer settings.

Category	Standard Specimens	EBC Shell
Printer/nozzle	Sovol SV06; 0.4 mm nozzle	Sovol SV06; 0.4 mm nozzle
Material	Ultrafuse^®^ PLA Tough	Ultrafuse^®^ PLA Tough
Nozzle/bed temp.	215 °C/60 °C	215 °C/60 °C
Layer height	0.20 mm	0.20 mm
Perimeters	2	4 (thin walls/fit surfaces)
Infill (pattern/density)	Rectilinear/100%	Rectilinear/100%
Print speed	60 mm/s	60 mm/s
Cooling	Fan on from layer 2	Fan on from layer 2
Supports	Disabled	Paint-on organic supports (internal only)
Slicer/version	PrusaSlicer 2.9.2	PrusaSlicer 2.9.2
Orientation	Cylinder upright; bar flat; beam arch up	Upright to preserve cavities/slots
Estimated time/material	Job: ~53 min; ~3.47 m (~10.4 g)	Part: ~30 min; ~1.32 m (~3.96 g)

**Table 5:** Thermal treatment stages.

Stage	Temperature Schedule	Notes
1	25 → 70 °C in 6 min	Programmable oven; parts fully embedded
2	70 → 100 °C in 9 min	Staged ramp to reduce thermal shock
3	100 → 120 °C in 5 min	Cross Tg; prepare for isothermal dwell
4	Hold 120 °C for 60 min	Constrained by NaCl or RTV (SVSIL-P25)
5	Cool to 25 °C (~30 min)	Remain embedded during cool-down

**Table 6:** Dimensional calculations.

Metric	Definition	Formula/Units
Δ (absolute change)	Deviation from CAD nominal	Δ = Measured − CAD [mm]
%Δ (relative change)	Percent deviation vs. CAD	%Δ = 100 × (Measured − CAD)/CAD [%]
Mean ± SD	Summary across replicates	Sample mean and SD (n = 6)

**Table 7:** Dimensional measurements of hollow cylinder specimens before and after annealing.

Description	CAD Dimensions (mm)	3D Print Dimensions (Mean) (mm)	Annealed in Salt		Annealed in Resin	
			Sample A-Black	Sample B-Natural	Sample C-Black	Sample D-Natural
Dext-Max	Ø20	Ø20.01	Ø19.76	Ø19.75	Ø20.18	Ø20.14
Dext-Min	Ø20	Ø19.96	Ø19.52	Ø19.43	Ø20.06	Ø20.02
Dint-Max	Ø16.8	Ø16.99	Ø16.68	Ø16.62	Ø17.16	Ø17.12
Dint-Min	Ø16.8	Ø16.94	Ø16.5	Ø16.5	Ø17.01	Ø17.03
Height (H)	25	25	25.34	25.39	25.01	25.08

**Table 8:** Percentual variation in dimensions average of hollow cylinder specimens after annealing in salt and resin.

Description	% Dimensions Variation Salt Average (%)	% Dimensions Variation Resin Average
Dext	−1.95	0.47
Dint	−2.50	0.47
Height (H)	1.46	0.18

**Table 9:** Dimensional evaluation of rectangular bar specimens.

Description	CAD Dimensions (mm)	3D Print Dimensions (Mean) (mm)	Annealed in Salt		Annealed in Resin	
			Sample A-Black	Sample B-Natural	Sample C-Black	Sample D-Natural
Height (H)-Max	5	5.03	5.15	5.23	5.03	5.18
Height-Min	5	5	5.08	5.14	5	5.04
Width (W)-Max	10	10.02	9.84	9.91	9.93	9.9
Width-Min	10	9.96	9.73	9.78	9.83	9.71
Length (L)	100	99.96	98.85	98.02	100.25	100.4

**Table 10:** Percentual variation in dimensions average of rectangular bar specimens after annealing in salt and resin.

Description	% Dimensions Variation Salt Average	% Dimensions Variation Resin Average
Height (H)	2.10	0.64
Width (W)	−2.22	−1.43
Length (L)	−1.14	0.12

**Table 11:** Dimensional evaluation of curved beam specimens.

Description	CAD Dimensions (mm)	3D Print Dimensions (Mean) (mm)	Annealed in Salt		Annealed in Resin	
			Sample A-Black	Sample B-Natural	Sample C-Black	Sample D-Natural
Height (H)-Max	1.6	1.57	1.6	1.6	1.55	1.56
Height-Min	1.6	1.53	1.55	1.5	1.52	1.49
Width (W)-Max	6	6.02	6.15	6.16	6.07	6.06
Width (W)-Min	6	5.97	6.08	6.06	6.03	5.99
Length	100	100.07	97.91	98.46	100.75	100.92

**Table 12:** Percentual variation in dimensions average of curved beam specimens after annealing in salt and resin.

Description	% Dimensions Variation Salt Average	% Dimensions Variation Resin Average
Height (H)	3.46	1.32
Width (W)	1.87	0.63
Length (L)	−1.93	0.71

## Data Availability

The original contributions presented in this study are included in the article; further inquiries can be directed to the corresponding author.

## Supplementary Materials

The following supporting information can be downloaded at: https://www.mdpi.com/article/10.3390/jfb16090334/s1, S1. Physical, mechanical (Table S1), and thermal (Table S2) properties: Table S1: Mechanical properties (ISO 527, ISO 178, ISO 179-2, ISO 180); Table S2: Thermal properties (ISO 75-2, ISO 306, ISO 11357, ISO 1133); S2. Three-dimensional Printing Orientation: Figure S1: Printing layout and orientation of test specimens (PrusaSlicer view); Figure S2: 3D printing layout and support strategy for the Easy Bone Collector Shell components; S3. Results—Mean Δ, SD Δ, Mean %Δ and SD %Δ are graphically presented in the article and below are presented values for all samples and sample types: Table S3. Hollow cylinder dimensions; Table S4. Rectangular bar dimensions; Table S5. Curved beam dimensions

## Abbreviations

The following abbreviations are used in this manuscript:

FDM	Fused Deposition Modeling
PLA	Polylactic Acid
EBC	Easy Bone Collector
CAD	Computer Aided Design
ABS	Acrylonitrile Butadiene Styrene
RTV	Room-Temperature Vulcanizing
PETG	Polyethylene Terephthalate Glycol-Modified
HTPLA	High-Temperature PLA
CNT	Carbon Nanotubes
FFF	Fused Filament Fabrication
DSC	Differential Scanning Calorimetry
SEM	Scanning Electron Microscopy
HDT	Heat Deflection Temperature
TMA	Thermo Mechanical Analysis
DMA	Dynamic Mechanical Analysis
SD	Standard Deviation
