# Supplementary material for: Sustainable Thermal Post-Processing of PLA 3D Prints: Increased Dimensional Precision and Autoclave Compatibility
Source: J Funct Biomater. 2025 Sep 8;16(9):334. doi: 10.3390/jfb16090334 (PMC12471281; doi:10.3390/jfb16090334)
Supplement: Supplementary file 1 [file jfb-16-00334-s001.zip › jfb-3830761-supplementary.pdf]

Supplementary Materials

S1. Physical, mechanical (Table S1), and thermal (Table S2) properties

The following physical, mechanical (Table S1), and thermal (Table S2) properties were provided in the extended technical data sheet:

1. **Filament Properties:**
- Diameter: 1.75 mm.
- Diameter tolerance: ±0.050 mm.
- Ovality: <0.050 mm.
- Colors: natural, black.
- Density (ISO 1183-1): 1215 kg/m³.

2. **Recommended Printing Parameters:**
- Nozzle temperature: 200–220 °C.
- Bed temperature: 50–70 °C.
- Nozzle diameter: ≥0.4 mm.
- Print speed: 40–300 mm/s.
- Bed material: glass.
- Build chamber: indirect heating.

3. **Annealing Recommendations:**
- Heating ramp: Room temperature → 120 °C in ~15 min.
- Holding: 120 °C for 30 min (longer for thicker parts).
- Cooling: Back to room temperature in ~15 min.

Note: Minor dimensional deviations may occur (e.g., up to +2% in thickness, –1% in width for ISO 527 Type 1A bars).

Table S1. Mechanical properties (ISO 527, ISO 178, ISO 179-2, ISO 180).

Property	XY Direction	XZ Direction	ZX Direction
Tensile strength	40 MPa	–	28 MPa
Elongation at break	74%	–	2.5%
Young's modulus	2672 MPa	–	2576 MPa
Flexural strength	73 MPa	75 MPa	51 MPa
Flexural modulus	2690 MPa	2410 MPa	2390 MPa
Impact strength (Charpy, notched)	18 kJ/m²	8.6 kJ/m²	2.5 kJ/m²
Impact strength (Charpy, unnotched)	33 kJ/m²	34 kJ/m²	10 kJ/m²

Table S2. Thermal properties (ISO 75-2, ISO 306, ISO 11357, ISO 1133).

Property	After Printing	Annealed
HDT A (1.8 MPa)	55 °C	65 °C
HDT B (0.45 MPa)	57 °C	94 °C
Vicat softening point at 50 N	59 °C	86 °C
Vicat softening point at 10 N	61 °C	157 °C
Glass transition temperature (Tg)	–	62 °C
Melting temperature	–	172 °C
MFR / MVR (210 °C, 2.16 kg)	5.68 g/10 min	–

Supplementary Materials

S2. Three-dimensional Printing Orientation

The slicing was performed in PrusaSlicer 2.9.2, and all models were arranged in a single print job for consistency. Figure 1 illustrates the orientation of the specimens on the print bed.

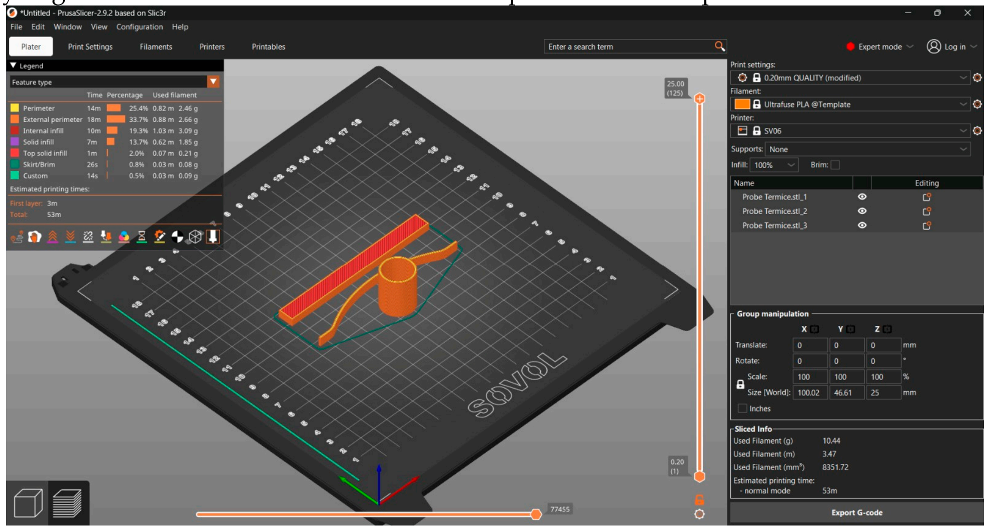

Figure S1: Printing layout and orientation of test specimens (PrusaSlicer view).

The cylindrical specimen was printed either vertically or upright in order to evaluate radial deformation and shrinkage along the build axis.

The rectangular bar was positioned flat on its 100 × 10 mm face to make it more sensitive to gravity drooping and thermal creep.

The curved beam was oriented with the arch facing upward to challenge the print with unsupported curvature. It was, therefore, ideal for post-processing studies on deformations.

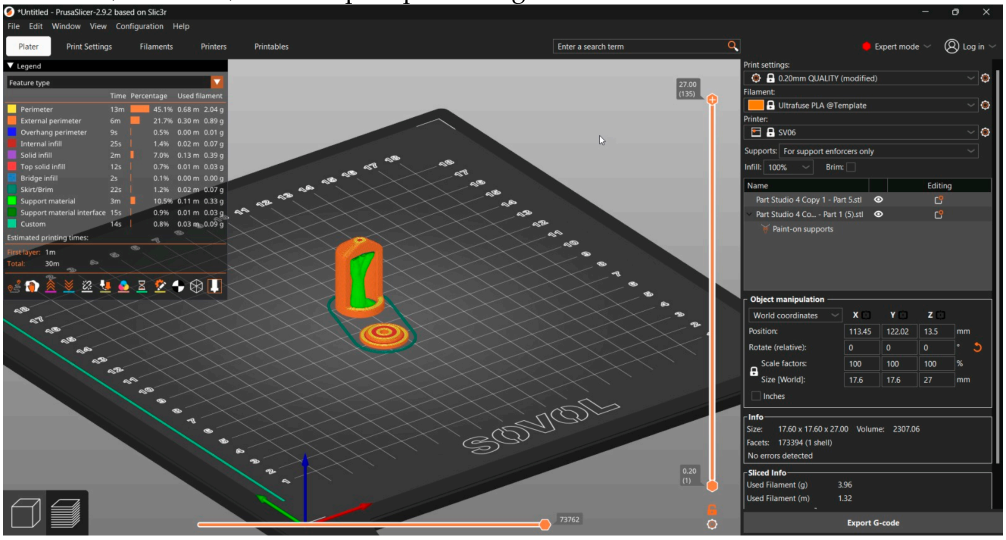

Figure S2: 3D printing layout and support strategy for the Easy Bone Collector Shell components.

The print arrangement and support setup are displayed in Figure 2. This part was expected to take 30 minutes to print, use 3.96 grams of filament, and require about 1.32 meters of extrusion. Support material

accounted for 10.5% of the volume, and the interface layers were designed to easily separate from the PLA body without compromising surface characteristics.

When complex contours and small radii were present, this arrangement allowed for the Easy Bone Collector shell's surface accuracy and dimensional tolerances to be maintained during printing and the ensuing heat treatment.

S3. Results - Mean Δ, SD Δ, Mean %Δ and SD %Δ are graphically presented in the article and below are presented values for all samples and sample types

Table S3. Hollow cylinder dimensions.

Dimensions	Mean Δ annealed in salt (mm) ± SD (n = 6) (mm)	Mean Δ annealed in resin (mm) ± SD (n = 6) (mm)	SD Δ annealed in salt (mm)	SD Δ annealed in resin (mm)	Mean %Δ annealed in salt ± SD (n = 6) (%)	Mean %Δ annealed in resin ± SD (n = 6) (%)	SD %Δ annealed in salt (%)	SD %Δ annealed in resin (%)
Dext - Max	-0.24	0.15	0.05	0.03	-1.21	0.74	0.27	0.17
Dext - Min	-0.53	0.03	0.06	0.02	-2.64	0.13	0.3	0.1
Dint- Max	-0.16	0.38	0.04	0.04	-0.92	2.25	0.25	0.23
Dint- Min	-0.34	0.24	0.04	0.03	-2.03	1.43	0.21	0.16
H	0.27	0.03	0.12	0.06	1.09	0.13	0.47	0.25

Table S4. Rectangular bar dimensions.

Dimensions	Mean Δ annealed in salt (mm) ± SD (n = 6) (mm)	Mean Δ annealed in resin (mm) ± SD (n = 6) (mm)	SD Δ annealed in salt (mm)	SD Δ annealed in resin (mm)	Mean %Δ annealed in salt ± SD (n = 6) (%)	Mean %Δ annealed in resin ± SD (n = 6) (%)	SD %Δ annealed in salt (%)	SD %Δ annealed in resin (%)
Height (H) – Max	0.18	0.1	0.06	0.05	3.67	2.03	1.18	1.05
Height - Min	0.11	0.04	0.03	0.04	2.23	0.73	0.7	0.88
Width (W) - Max	-0.1	-0.08	0.08	0.03	-0.98	-0.78	0.8	0.34
Width - Min	-0.24	-0.2	0.04	0.06	-2.37	-1.97	0.37	0.59
Length (L)	-1.41	0.42	0.48	0.13	-1.41	0.42	0.48	0.13

Supplementary Materials

Table S5. Curved beam dimensions.

Dimensions	Mean Δ annealed in salt (mm) ± SD (n = 6) (mm)	Mean Δ annealed in resin (mm) ± SD (n = 6) (mm)	SD Δ annealed in salt (mm)	SD Δ annealed in resin (mm)	Mean %Δ annealed in salt ± SD (n = 6) (%)	Mean %Δ annealed in resin ± SD (n = 6) (%)	SD %Δ annealed in salt (%)	SD %Δ annealed in resin (%)
Height (H) – Max	0.01	-0.03	0.03	0.02	0.42	-1.88	1.66	1.12
Height - Min	-0.07	-0.08	0.02	0.02	-4.48	-5	1.45	1.19
Width (W) - Max	0.17	0.04	0.03	0.03	2.83	0.61	0.49	0.47
Width - Min	0.06	-0.01	0.02	0.02	1.08	-0.17	0.38	0.35
Length (L)	-1.93	0.96	0.23	0.18	-1.93	0.96	0.23	0.18
